# Isolation and Molecular Characterization of Atypical Porcine Pestivirus Emerging in China

**DOI:** 10.3390/v15112149

**Published:** 2023-10-25

**Authors:** Hao Song, Xiaowei Gao, Yanhui Fu, Jing Li, Gaocheng Fan, Lina Shao, Jiaoer Zhang, Hua-Ji Qiu, Yuzi Luo

**Affiliations:** State Key Laboratory for Animal Disease Control and Prevention, Harbin Veterinary Research Institute, Chinese Academy of Agricultural Sciences, 678 Haping Road, Harbin 150069, China; songhao1109@163.com (H.S.); gaoxiaowei2022@126.com (X.G.); fuyanhui20190301@163.com (Y.F.); lijing36743@163.com (J.L.); fgc000630@163.com (G.F.); 13634615691@163.com (L.S.); jiaoerz@126.com (J.Z.)

**Keywords:** atypical porcine pestivirus, prevalence, virus isolation, characterization

## Abstract

Atypical porcine pestivirus (APPV) is a recently discovered and very divergent species of the genus *Pestivirus* within the family *Flaviviridae*, which causes congenital tremor (CT) in newborn piglets. In this study, an APPV epidemiological investigation was conducted by studying 975 swine samples (562 tissue and 413 serum samples) collected from different parts of China from 2017 to 2021. The results revealed that the overall positive rate of the APPV genome was 7.08% (69/975), among which 50.7% (35/69) of the samples tested positive for one or more other common swine viruses, especially porcine circovirus type 2 (PCV2) with a coinfection rate of 36.2% (25/69). Subsequently, a novel APPV strain, named China/HLJ491/2017, was isolated in porcine kidney (PK)-15 cells for the first time from a weaned piglet that was infected with both APPV and PCV2. The new APPV isolate was confirmed by RT-PCR, sequencing, immunofluorescence assay, and transmission electron microscopy. After clearing PCV2, a pure APPV strain was obtained and further stably propagated in PK-15 cells for more than 30 passages. Full genome sequencing and phylogenetic analysis showed that the China/HLJ491/2017 strain was classified as genotype 2, sharing 80.8 to 97.6% of its nucleotide identity with previously published APPV strains. In conclusion, this study enhanced our knowledge of this new pestivirus and the successful isolation of the APPV strain provides critical material for the investigation of the biological and pathogenic properties of this emerging virus, as well as the development of vaccines and diagnostic reagents.

## 1. Introduction

Pestiviruses, which belong to the genus *Pestivirus* of the family *Flaviviridae*, are highly variable RNA viruses that can cause economically relevant diseases in livestock animals and wildlife species. Classical swine fever virus (CSFV, *Pestivirus A*), bovine viral diarrhea virus 1 (BVDV-1, *Pestivirus B*), bovine viral diarrhea virus 2 (BVDV-2, *Pestivirus C*), and border disease virus (BDV, *Pestivirus D*) are considered the so-called classical pestiviruses. In the last two decades, genetically diverse pestiviruses, such as pronghorn pestivirus [1], Bungowannah virus [2], and rat pestivirus [3], have been identified. Recently, members of the genus *Pestivirus* have been classified into species *Pestivirus A* to *K* by the International Committee on Taxonomy of Viruses [4,5]. 

Atypical porcine pestivirus (APPV) is a recently discovered virus that is classified as *Pestivirus K* of the genus *Pestivirus*, which was first identified in the United States of America (USA) in 2015 [6]. Since its discovery, APPV has been shown to be associated with congenital tremor (CT) type A-II in newborn piglets via the inoculation of pregnant sows with APPV-infected animal materials, which induced neurological disorders in newborn piglets, reduced reproductive performance of sows, and increased preweaning mortality [7,8]. APPV is a highly variable RNA virus that has a single-stranded, positive-sense RNA genome that is approximately 11 to 12 kb in size and contains a large open reading frame (ORF) surrounded by 5′- and 3′-untranslated regions (UTRs) [6,9]. The ORF is translated into a polyprotein encoding 3635 amino acids that is later cleaved into individual proteins, including four structural proteins (C, E^rns^, E1, and E2) and eight non-structural proteins (N^pro^, p7, NS2, NS3, NS4A, NS4B, NS5A, and NS5B) [6]. The envelope glycoprotein E2 of pestiviruses has been shown to induce strong neutralizing antibodies and thus, is an ideal target for diagnostic kits and vaccines. Interestingly, in contrast to those of CSFV and BVDV, the E2 protein of APPV lacks two N-terminal domains, resulting in an E2 protein (241 aa) that is significantly different from those of other pestiviruses (373 to 378 aa) [10]. Since the E2 protein of pestiviruses is responsible for mediating virus entry into cells, this deletion may cause differences in viral entry between APPV and other pestiviruses.

Since it was first reported in the USA in 2015 [6], APPV has been detected in domestic pigs and wild boars with or without CT on different continents, including the Americas, Europe, and Asia [6,11,12,13]. Mortality has been reported to be as high as 30% among APPV-infected piglets [14], seriously affecting the pig industry. In a study including 1460 sera from apparently healthy pigs in Germany, Italy, Serbia, United Kingdom, Switzerland, and China, the overall prevalence of the APPV genome was 8.9%, varying from 2.3% (2/86) from United Kingdom to 17.5% (35/200) from Italy [11]. The APPV genome was also detected in 11/219 samples (5%) from Mainland China, but the sampling areas were not clear. In 2016, APPV infection was first reported in piglets with CT in Guangdong Province in China [12]. Subsequently, CT cases in piglets caused by APPV have been reported in other regions of China, including Guangxi, Anhui, Hubei, and Henan [15,16,17,18,19]. The APPV strains from China displayed high sequence variability, ranging from 83 to 95% [20,21]. Although several APPV clusters have been proposed, the origin and evolution of APPV in China are still unclear and a continuous epidemiological survey covering more regions is necessary.

Another issue is that APPV propagation is considered to be highly inefficient in primary porcine kidney cells and continuous porcine cell lines (PK-15, IBRS-2, SK6, and ST) [6,12]. Therefore, to date, only one APPV strain, Ger-NRW_L277, has been successfully isolated and stably propagated on embryonic porcine kidney epithelial cells (SPEV cells) by a German research team [22]. A Chinese research group reported the isolation of an APPV strain (China/HeN01/2018) in SPEV cells [18], but detailed characterization of the strain has not yet been described. 

In this study, the prevalence and genetic characteristics of APPV in domestic pigs in different regions of China were investigated. Further, a novel APPV strain, named China/HLJ491/2017, was isolated for the first time in PK-15 cells from a weaned piglet and its molecular and biological properties were characterized.

## 2. Materials and Methods

### 2.1. Epidemiological Investigation

A total of 975 clinical porcine samples (562 tissues and 413 sera from healthy or diseased pigs) were tested for APPV using RT-PCR and RT-qPCR, as described by Postel et al. [23] and Liu et al. [24]. These samples were collected for passive surveillance by farmers or local veterinarians during 2017 to 2021 from 138 pig herds in different provinces of China, including Heilongjiang (529 samples from 93 pig farms), Jilin (48 samples from 8 pig farms), Inner Mongolia (54 samples from 5 pig farms), Hubei (47 samples from 5 pig farms), Shandong (38 samples from 6 pig farms), Liaoning (59 samples from 9 pig farms), Beijing (86 samples from 6 pig farms), Henan (62 samples from 3 pig farms), and Jiangsu (52 samples from 3 pig farms). Subsequently, the samples were sent to our laboratory for diagnosis, and stored at −80 °C. All APPV-positive samples were further tested as described previously for porcine circovirus type 2 (PCV2) [25], CSFV [26], PRRSV [27], pseudorabies virus (PRV) [28], and porcine epidemic diarrhea virus (PEDV) [29].

### 2.2. RT-PCR and Genome Sequencing

The total RNA was extracted from all samples using the Simply P Total RNA Extraction Kit (BioFlux, Hangzhou, China), according to the manufacturer’s protocols. Reverse transcription was performed using a final volume of 20 μL, containing 6 μL of RNA template, 4 μL of dNTPs (10 mM each), 1 μL of 9-random hexamers, 1 μL of reverse transcriptase XL (AMV), 4 μL of 5 × AMV buffer, and 0.5 μL of RNase Inhibitor (TaKaRa, Beijing, China), and the samples were then incubated at 42 °C for 1 h. Subsequently, PCR was conducted using *Ex Taq* HS DNA polymerase (TaKaRa, Beijing, China), in accordance with the manufacturer’s instructions. The forward primer APPV_5030-fw (5′-CCC AGG CAA TAC CTC ACA AC-3′) and reverse primer APPV_5469-rev (5′-CCC CTT TTT GGT TCC TCT CC-3′) were used to amplify the partial NS3 gene of APPV (440 bp), according to a previous study [23]. The E2 gene was amplified according to previously published nested PCR primers [30]. The PCR conditions for the NS3 or E2 gene amplification consisted of an initial denaturation for 5 min at 94 °C, followed by 35 amplification cycles (denaturation for 30 s at 94 °C, annealing for 30 s at 56 °C, and extension for 45 s at 72 °C), and a final extension for 10 min at 72 °C. The PCR products were purified with the Gel Extraction Kit (Omega, Honolulu, HI, USA) and sent to RuiBiotech Co. Ltd. (Harbin, China) for sequencing.

### 2.3. Cell Culture, Virus Isolation, and Immunofluorescence Assay (IFA)

Porcine kidney (PK)-15 cells were cultured in Dulbecco’s modified Eagle’s medium (DMEM) (Gibco, Beijing, China), which was supplemented with 10% fetal bovine sera (FBS) (Sigma-Aldrich, St. Louis, MI, USA), 100 μg/mL of streptomycin, and 100 IU/mL of penicillin, at 37 °C in 5% CO_2_. APPV genome-positive tissues were homogenized and resuspended in 10 volumes of DMEM. Subsequently, the serum or the homogenate of the tissues was centrifuged and passed through a 0.45-µm filter (Millipore, Burlington, MA, USA) before being inoculated into PK-15 cells in 6-well plates. The cells were incubated at 37 °C in 5% CO_2_ for 72 h and then the cultures were harvested and subjected to three freeze–thaw cycles. Next, the supernatant was inoculated into the new confluent monolayers of the PK-15 cells. At each passage, the cultures were verified by RT-PCR and IFA. The IFA was conducted as previously described, with slight modifications [31]. In brief, PK-15 cells were seeded in 96-well plates and inoculated with the cultures of each passage mentioned above. The cells were incubated at 37 °C in 5% CO_2_ for 72 h and then fixed with pre-chilled absolute ethanol at 4 °C for 30 min. The anti-APPV-E2 monoclonal antibody (MAb) 6E2 prepared by our laboratory (diluted 1:200 in 5% bovine serum albumin) was used as the primary antibody and was incubated with the cells at 37 °C for 2 h. The cells were washed five times with PBS, containing 0.05% Tween 20 (PBST), and then incubated with FITC-labeled anti-mouse IgG (Invitrogen, Carlsbad, CA, USA) for 2 h at 37 °C. After being washed five times with PBST, the cells were examined using a fluorescence microscope (Nikon TE200, Tokyo, Japan). The viral titers of the cultures were calculated as described by Reed and Muench [32].

### 2.4. Transmission Electron Microscopy (TEM)

After centrifuge at 2000× *g* for 30 min, the supernatants of the APPV-infected PK-15 cells were collected and passed through a 0.22-µm filter, followed by centrifuge at 100,000× *g* for 3 h in an SW32Ti rotor (Beckman, Brea, CA, USA). The virus pellet was re-suspended in 200 μL of PBS buffer and then processed for negative staining with 2% phosphotungstic acid (pH 7.0). Subsequently, the samples were observed on an H-7650 TEM (Hitachi, Tokyo, Japan). The APPV-infected PK-15 cells were fixed in 2.5% glutaraldehyde and cell ultra-thin sections were processed for electron microscopy as described previously [33].

### 2.5. Complete Genome Sequencing

In total, 10 pairs of primers were designed using SnapGene 4.1.9 software and were used to amplify the complete genome of APPV (Table S1). The full genome amplification conditions consisted of initial denaturation for 5 min at 95 °C, followed by 35 cycles (denaturation for 30 s at 94 °C, annealing for 30 s at 56 °C, and extension for 1 min 45 s at 72 °C), and a final extension for 10 min at 72 °C. The full-length genomic sequence of each isolate was generated by assembling overlapping fragments using the SeqMan module in DNAStar Lasergene 7. The PCR products were purified with the Gel Extraction Kit (Omega, Honolulu, HI, USA) and cloned into the pMD18-T vector (TaKaRa, Beijing, China) and then the mixture was transformed into *E. coli* (DH5α) competent cells. The selected positive colonies were cultured and sent to RuiBiotech Co. Ltd. (Harbin, China) for sequencing.

### 2.6. Sequence Alignment and Phylogenetic Analysis 

Phylogenetic analysis was performed by comparing the nucleotide sequences of the APPV strains obtained in the present study (Table 1) to the APPV sequences published in the GenBank database. Multiple sequence alignments and sequence similarity calculations were performed using the MegAlign module in DNAStar Lasergene 7. Phylogenetic trees were constructed by applying the neighbor-joining method using MEGA 7.0 software, with 1000 bootstrap replicates [34].

### 2.7. Virus Growth Curve

The monolayers of PK-15 cells seeded in 24-well plates were infected with APPV at a multiplicity of infection (MOI) of 0.1 and were incubated for 2 h at 37 °C in 5% CO_2_ to allow virus attachment. Thereafter, the inoculum was replaced with pre-warmed fresh medium and the cultures were harvested at 12, 24, 36, 48, 60, 72, and 84 h post-inoculation (hpi). After three freeze–thaw cycles, the lysates were used to determine the viral titers in the PK-15 cells. The mean values and standard deviations of three independent experiments were then calculated.

### 2.8. Statistical Analysis

The data were analyzed using GraphPad Prism 8.3.0 software. Differences were considered significant at *p* < 0.05 (*).

## 3. Results

### 3.1. Prevalence of APPV among Domestic Pigs in China

To investigate the prevalence of APPV in pig herds in China, a total of 975 swine samples (562 tissue and 413 serum samples) were collected from nine provinces across China from 2017 to 2021 and tested using RT-qPCR. The results showed that the overall positive rate of the APPV genome was 7.08% (69/975). To explore the temporal trends of APPV prevalence, surveillance data were further analyzed. The results showed that the positive rate of APPV was 4.74 to 8.11% from 2017 to 2021 (Table 2). Among the different types of samples tested, the positive rate of tissues was 6.94% (39/562), while that of sera was 7.26% (30/413) (Table 3).

To investigate the coinfection status of APPV-circulating pig herds, the APPV-positive samples were also tested for PCV2, PRRSV, PRV, PEDV, and CSFV. The results showed that 50.7% (35/69) of the APPV-positive samples also tested positive for other common swine viruses, including PCV2 (25/69; 36.2%), PRV (7/69; 10.14%), CSFV (5/69; 7.25%), PEDV (4/69; 5.80%), and PRRSV (2/69; 2.90%) (Table 4).

### 3.2. Genetic Characterization of APPV Isolates

**Figure 1:**
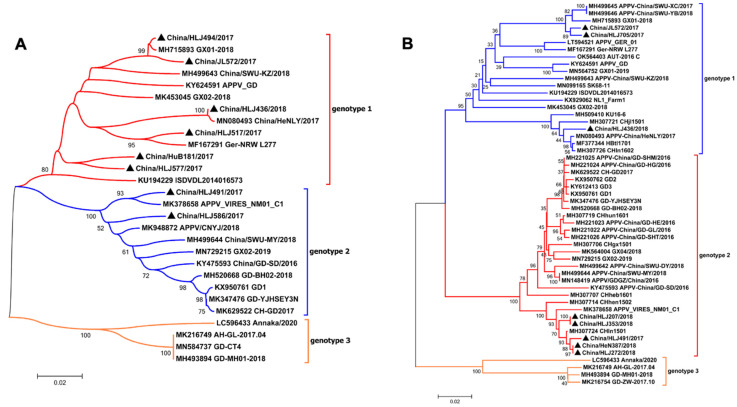
Phylogenetic analysis of APPV strains from different regions of China. The phylogenetic trees were based on the partial NS3 nucleotide sequences (**A**) or complete E2 nucleotide sequences (**B**) of APPV. The phylogenetic trees were generated using MEGA 7.0 software with the neighbor-joining method with 1000 bootstrap replicates. The scale bar indicates the corrected genetic distances. The percent bootstrap support is indicated by the value at each node (values < 50% were omitted). The black triangles indicate sequences that were obtained in this study.

To identify and characterize the different APPV isolates, the APPV-positive samples were further subjected to the amplification of the partial NS3 gene and the complete E2 gene, using RT-PCR for sequencing. In total, 8 NS3 and E2 gene nucleotide sequences were obtained from 13 isolates, which were submitted to GenBank with the accession numbers OP617199 and OQ575347 to OQ575361 (Table 1). Subsequently, phylogenetic analysis was conducted based on the nucleotide sequences of the partial NS3 gene with 20 APPV reference strains and those of the complete E2 gene with 45 APPV strains. As shown in Figure 1A, six isolates (China/HuB181/2017, China/HLJ436/2018, China/HLJ494/2017, China/HLJ517/2017, China/JL572/2017, and China/HLJ577/2017) were classified as genotype 1, while two isolates (China/HLJ491/2017 and China/HLJ586/2017) were classified as genotype 2. Similarly, as shown in Figure 1B, three isolates (China/JL572/2017, China/HLJ705/2017, and China/HLJ436/2018) were identified as genotype 1, while five isolates (China/HLJ491/2017, China/HLJ207/2018, China/HLJ272/2018, China/HLJ353/2018, and China/HeN387/2018) were classified as genotype 2.

### 3.3. Virus Isolation and Characterization

We attempted to isolate APPV in continuous porcine cell lines and a new APPV strain, named China/HLJ491/2017, was successfully isolated for the first time in PK-15 cells from an APPV-positive weaned piglet that was coinfected with PCV2. After five blind passages in PK-15 cells, the virus cultures of each passage tested positive for both APPV and PCV2, according to RT-qPCR and qPCR, respectively. To obtain the pure APPV strain, the virus cultures from the 10th passage were treated with anti-PCV2 sera prior to the next passage and the cultures were confirmed to be PCV2-free from the 17th passage, according to PCV2 qPCR. The cultures were also tested negative for CSFV and PRV.

**Figure 2:**
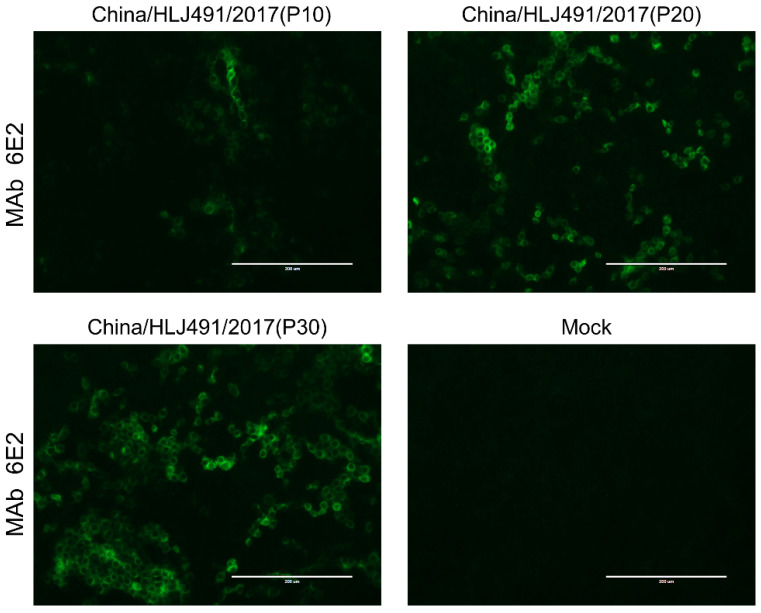
Isolation and identification of the APPV China/HLJ491/2017 strain. The APPV China/HLJ491/2017 strain can stably propagate in PK-15 cells. PK-15 cells were inoculated with the 10th, 20th, and 30th passages of China/HLJ491/2017 at an MOI of 0.1 and incubated for 72 h and were subsequently identified by IFA. The scale bar is 200 μm.

**Figure 3:**
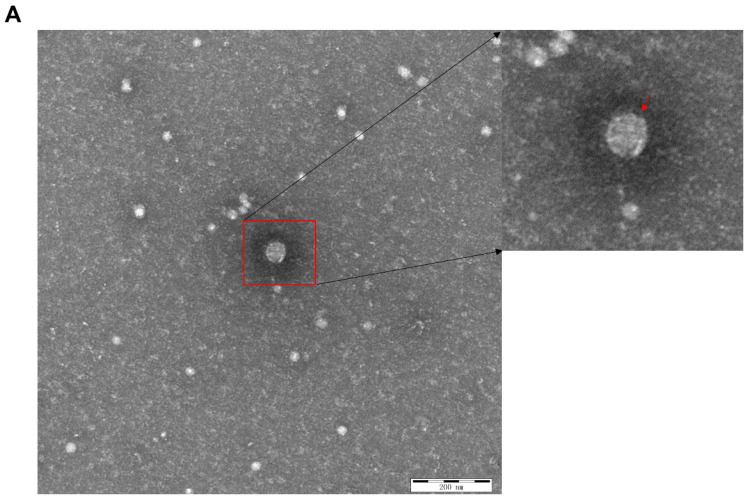

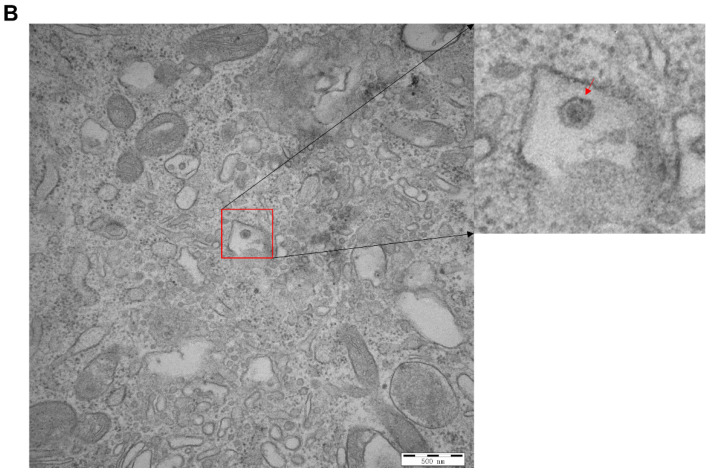
Electronic microscopy examination (**A**) and an ultrathin section (**B**) of the China/HLJ491/2017 strain, showing that the virions (indicated by a box or arrow) were around 50 nm in diameter with icosahedral nucleocapsids.

The APPV China/HLJ491/2017 strain was further passaged in PK-15 cells for more than 30 passages and was verified by IFA. The results showed that the virus cultures in different generations reacted specifically with anti-APPV-E2 MAb, indicating that the isolated APPV strain could be stably propagated in PK-15 cells (Figure 2). Electron microscopy showed that APPV virions in ultra-thin sections were enveloped and spherical, with a diameter of 50 nm, consistent with the morphology of CSFV (Figure 3). 

### 3.4. Full Genome Characterization of the Isolated APPV Strain

**Figure 4:**
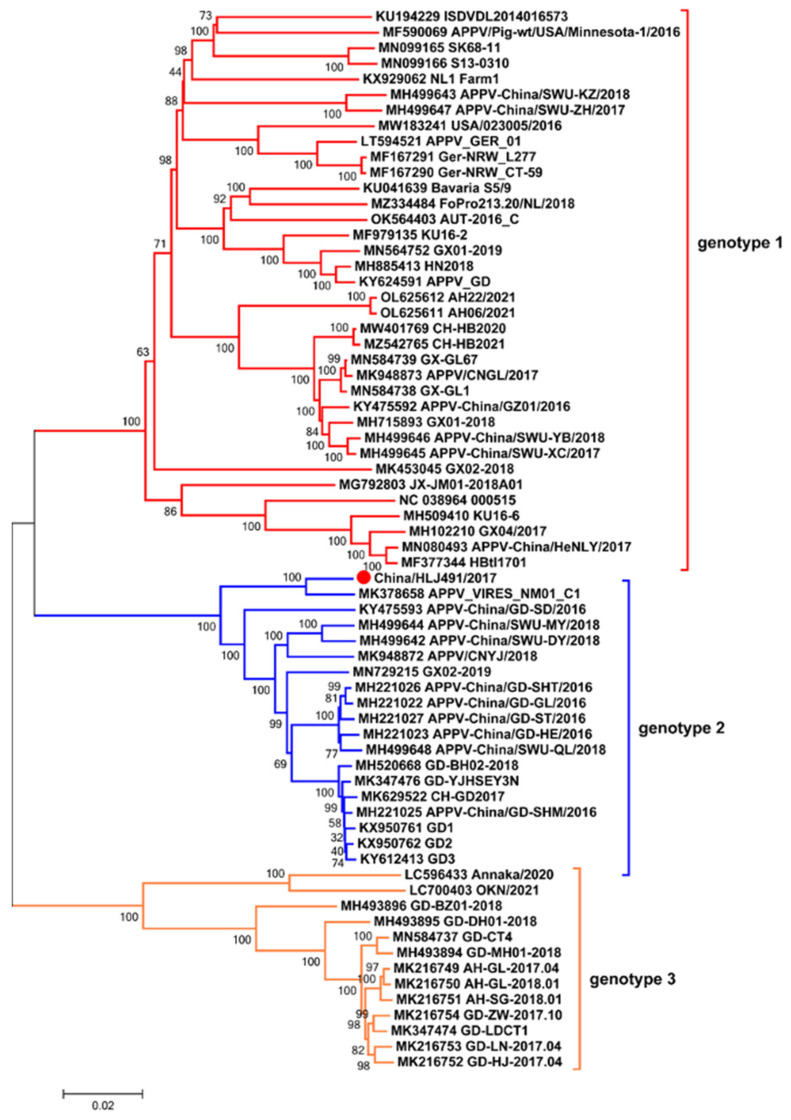
Phylogenetic tree based on the polyprotein nucleotide sequences of the China/HLJ491/2017 strain. The China/HLJ491/2017 strain obtained in this study is indicated by the red circle.

To further analyze the genomic characteristics of the APPV China/HLJ491/2017 strain, the complete genome sequence was obtained by the Sanger’s sequencing and was subsequently submitted to GenBank (accession number OQ032517). The complete genome consists of 11,511 nucleotides (nt), including 5′UTR (326 nt), ORF (10,908 nt), and 3′UTR (277 nt). The phylogenetic tree based on the sequence of the polyprotein was constructed together with 67 reference strains from China and other countries. The results showed that the APPV strains were clustered into three distinct genotypes (genotypes 1 to 3). The APPV strains isolated from China were assigned to all genotypes, whereas all APPV strains from North American and European countries belonged to genotype 1 (Figure 4). The newly isolated APPV China/HLJ491/2017 strain belonged to genotype 2 and formed a new branch with the APPV_VIRES_NM01_C1 strain. A comparison of the complete polyprotein sequences showed that the China/HLJ491/2017 strain shared 80.8 to 97.6% of its nucleotide identity at the genomic level and 90.9 to 98.9% of its amino acid identity with the polyprotein of 24 APPV strains from various countries (Table S2).

### 3.5. Replication Kinetics of the Isolated APPV Strain

**Figure 5:**
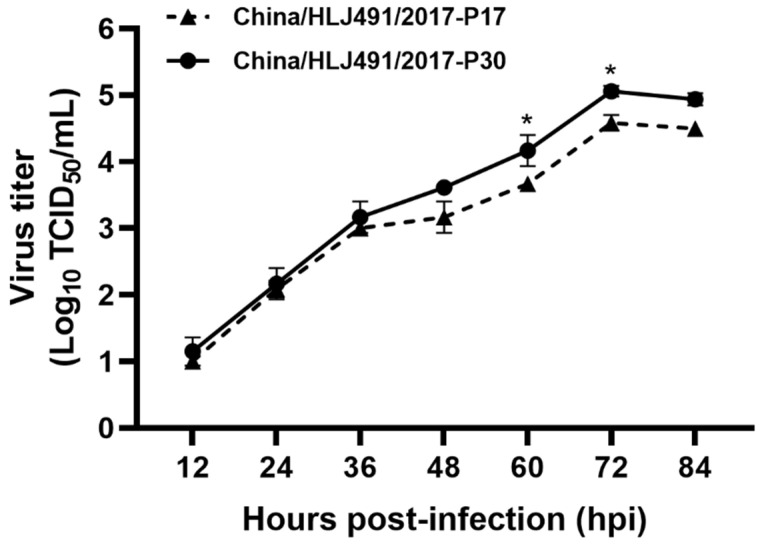
Growth kinetics of the China/HLJ491/2017 strain in PK-15 cells. PK-15 cell monolayers were infected with the 17th and 30th passage of the China/HLJ491/2017 strain at an MOI of 0.1 and were harvested at 12, 24, 36, 48, 60, 72, and 84 hpi. Viral titers were determined in PK-15 cells using IFA in triplicate and expressed as TCID_50_/mL. The mean values with standard deviations of three independent experiments were calculated. * p < 0.05.

To determine the replication characteristics of APPV, the growth kinetics of the China/HLJ491/2017 strain were tested at passages 17 (when it first separated from PCV2) and 30. As shown in Figure 5, the virus grew efficiently in PK-15 cells. Interestingly, the 30th passage of the China/HLJ491/2017 strain exhibited significantly higher replication levels at 60 and 72 hpi (*p* < 0.05) than the 17th passage.

## 4. Discussion

CT is a neurological disease that mainly affects newborn piglets. Piglets with CT often display mild to severe tremors in their head and body due to the demyelination of the central nervous system [35]. According to histopathological lesions in the central nervous system, CT has been divided into six different subtypes (AI–V and B). Type A-II CT has been shown to be associated with APPV infection [7,8]. Since APPV was discovered, many countries have reported cases of the virus. According to recent APPV epidemiology studies on apparently healthy pigs in Europe, America, and Asia, the positive rate of APPV ranges from 0.75 to 22% [12,23,36,37,38,39,40], suggesting that APPV is probably widespread across the global swine industry. Epidemiological investigations and the isolation of diverse APPV strains remain the focus of research.

In this study, a total of 975 samples from pig farms in different regions of China from 2017 to 2021 were tested for the APPV genome. The overall prevalence of APPV was 7.08% (69/975) and the positive rate revealed an increasing trend over time from 4.74 to 8.11%. Notably, a higher number of samples (803) were obtained from 2017 to 2018, before the emergence of African swine fever (ASF) in China in August 2018 [41]. This was consistent with a previous study, which found that the outbreaks of ASF in China resulted in a rapid decline in pig production [42]. In addition, most APPV-positive samples were from Heilongjiang Province, possibly due to the larger proportion of samples from this area. Some studies have shown that the positive rate of the APPV genome in wild boars across Europe is 0.23 to 19%, indicating that wild boars could be an important reservoir for this virus [43,44,45]. To date, there has been no research on the epidemiology of APPV among wild boar populations in China, the monitoring of which is necessary in the future.

The coinfection status of APPV and other common swine viruses was also investigated in this work. It has been reported that pigs are frequently coinfected with APPV and other swine viruses, such as CSFV, PRRSV, PCV2, etc. [6,16,39]. Our study indicated that the overall coinfection rate of APPV with other swine viruses (PCV2, PRV, CSFV, PEDV, and PRRSV) was as high as 50.7% (35/69), within which the coinfection rate with PCV2 was the highest, reaching 36.2% (25/69). Carrying these viruses may impair the immunity of piglets and enhance their susceptibility to APPV infection. Possatti et al. (2018) also reported the coinfection with APPV and porcine teschovirus (PTV) in pig farms in Brazil [46], which resulted in more severe clinical signs and increased mortality among piglets. Therefore, the roles of PCV2 and/or other swine viruses in CT caused by APPV need to be investigated further, which will contribute to our understanding of the pathogenesis of APPV.

It has been shown that APPV cannot propagate efficiently in primary porcine kidney cells or continuous porcine cell lines, such as PK-15, IBRS-2, SK6, and ST [6,12]. So far, only Becher’s group has successfully isolated an APPV strain of genotype 1 stably propagating in SPEV cells [22]. Based on the isolated APPV strain, they developed a virus neutralization test (VNT) [47] and also proved that the porcine complement regulatory protein CD46 is a major receptor for APPV [48]. In this study, we successfully isolated an APPV strain (China/HLJ491/2017) in PK-15 cells for the first time from a weaned piglet that was infected with APPV and PCV2. The new isolate of APPV was fully characterized using RT-PCR, full genome sequencing, IFA, and TEM assays. In addition, significant differences were observed in viral replication levels between the 30th and 17th generations at 60 and 72 hpi (*p* < 0.05). Subsequently, the complete genomes of the 17th and 30th generations were sequenced and various amino acid mutations were found, notably within the E2 protein. This indicated that the amino acid mutations in the virus could enhance viral replication in cells. The molecular mechanisms underlying these increased viral replication levels are worth exploring in the future.

It is the first report that APPV (China/HLJ491/2017 strain) was successfully isolated in PK-15 cells from coinfected samples, although other viruses isolated from coinfected samples have been previously described. For instance, Mi et al. (2020) isolated porcine astrovirus 5 (PAstV5) from a pig tissue sample that was tested positive for CSFV and suggested that coinfection with CSFV could have been a key factor in its successful isolation due to the suppression of type I interferon responses, which significantly enhance PAstV5 replication in PK-15 cells [49]. Xing et al. (2020) successfully isolated a Getah virus (GETV) from two CSFV-positive samples [50]. Previous studies have shown that PCV2 can impair host antiviral immunity, thus promoting the susceptibility and pathogenicity of other viruses in pigs [51,52,53,54]. Further research is required to investigate whether coinfection with PCV2 affects APPV replication. APPV is currently classified into three genotypes, of which genotype 1 has been reported on multiple continents, while genotypes 2 and 3 have only been described in Asia [40,55,56]. The phylogenetic trees based on the NS3 and E2 genes showed that the new APPV isolates in this study belonged to genotypes 1 and 2. It is noteworthy that some new isolates from China formed a new branch in genotype 2 based on the E2 gene, suggesting the emergence of a new sub-genotype. In addition, sequence alignment and phylogenetic analysis based on the complete ORF showed that the APPV China/HLJ491/2017 strain belonged to genotype 2 and formed a new branch with the APPV_VIRES_NM01_C1 strain from Inner Mongolia. The new strain shares 80.8 to 97.6% of its nucleotide identity with previously published APPV strains and is most closely related to the APPV_VIRES_NM01_C1 strain (97.6%), indicating that the APPV genome shows a high genetic diversity.

In conclusion, our study reported the successful isolation and stable propagation of a novel APPV strain, China/HLJ491/2017, in PK-15 cells and full analyzed the biological characteristics of the virus. Next, we will evaluate the pathogenicity of the virus in pigs and conduct diagnostics and vaccine development studies for this novel strain in order to provide effective control strategies for APPV infection.

## Figures and Tables

**Table 1:** New APPV isolates from China that were identified in this study.

No.	Isolate	Gene	GenBank Accession No.	Year	Genotype	Province
1	China/HuB181/2017	NS3	OQ575347	2017	1	Hubei
2	China/HLJ494/2017	NS3	OQ575349	2017	1	Heilongjiang
3	China/HLJ517/2017	NS3	OQ575350	2017	1	Heilongjiang
4	China/HLJ577/2017	NS3	OQ575352	2017	1	Heilongjiang
5	China/HLJ586/2017	NS3	OQ575353	2017	2	Heilongjiang
6	China/HLJ491/2017	NS3/E2	OQ575348/OP617199	2017	2	Heilongjiang
7	China/JL572/2017	NS3/E2	OQ575351/OQ575355	2017	1	Jilin
8	China/HLJ436/2018	NS3/E2	OQ575354/OQ575361	2018	1	Heilongjiang
9	China/HLJ705/2017	E2	OQ575356	2017	1	Heilongjiang
10	China/HLJ207/2018	E2	OQ575357	2018	2	Heilongjiang
11	China/HLJ272/2018	E2	OQ575358	2018	2	Heilongjiang
12	China/HLJ353/2018	E2	OQ575359	2018	2	Heilongjiang
13	China/HeN387/2018	E2	OQ575360	2018	2	Henan

**Table 2:** Prevalence of APPV in pig herds in China by year.

Year	Total Samples	Positive Samples	Prevalence (%)
2017	211	10	4.74
2018	592	47	7.94
2019	58	4	6.90
2020	40	2	5.00
2021	74	6	8.11
Total	975	69	7.08

**Table 3:** Prevalence of APPV in pig herds in China by sample type.

Sample Type	Total Samples	Positive Samples	Prevalence (%)
Tissues	562	39	6.94
Sera	413	30	7.26
Total	975	69	7.08

**Table 4:** Coinfection of APPV and other common swine viruses.

Coinfection (*n* = 69)	Positive Samples	Percentage of Positive Samples (%)
APPV only	34	49.3
APPV + PCV2	18	26.1
APPV + PRV	4	5.80
APPV + CSFV	1	1.45
APPV + PEDV	4	5.80
APPV + PRRSV	1	1.45
APPV + PCV2 + CSFV	3	4.35
APPV + PCV2 + PRV	2	2.90
APPV + PCV2 + PRRSV	1	1.45
APPV + PCV2 + CSFV + PRV	1	1.45
APPV + any other swine virus	35	50.7

## Data Availability

The data that support the findings of this study are openly available from the NCBI at https://www.ncbi.nlm.nih.gov/nuccore/, accession numbers OQ032517, OP617199, and OQ575347 to OQ575361.

## Supplementary Materials

The following supporting information can be downloaded at https://www.mdpi.com/article/10.3390/v15112149/s1, Table S1: PCR primers used for full genome analysis of APPV. Table S2: Differences in sequence identity between the APPV China/HLJ491/2017 strain isolated in this study and representative APPV strains published in GenBank (nt/aa%).
