# Supplementary material for: Isolation and Molecular Characterization of Atypical Porcine Pestivirus Emerging in China
Source: Viruses. 2023 Oct 25;15(11):2149. doi: 10.3390/v15112149 (PMC10675531; doi:10.3390/v15112149)
Supplement: Supplementary file 1 [file viruses-15-02149-s001.zip › viruses-2667258-supplementary.pdf]

Table S1. PCR primers used for whole genome analysis of the APPV.

Primer	Sequence (5′→3′)	Size (bp)
APPV-1F	AGCTCTGCAGTACGTGGGGG	1177
APPV-1R	AGGCACATCACCAGGGCTATGT	
APPV-2F	GAAGGGGATGTAGTGAAAGT	1048
APPV-2R	CGGGGAGCTCTACTGTGATAT	
APPV-3F	TACGGAACTGAATGGTTGGAAC	1211
APPV-3R	AAGGCCACAAGACTTGCAGT	
APPV-4F	CACCCTAACTCTATGGAAGAT	1317
APPV-4R	GTGACGTGTTTGGCACTGCT	
APPV-5F	GTCCACTCGACAAGGAAACT	1367
APPV-5R	CTTACCAGTGAGGTGACAAT	
APPV-6F	TTGACGTGGTCATAGACACT	1423
APPV-6R	ACCACCACTGCTGATTCCAT	
APPV-7F	CCGATTACTTGCCCTATGCT	1398
APPV-7R	GATGGCATTCGTAGCCCTCT	
APPV-8F	GTGCTAAGTCCCTGCTACCT	1297
APPV-8R	AGGCCGCCCACATTTTTTCGT	
APPV-9F	AAACACCTCTGGCCCAAAAT	1225
APPV-9R	AGTTGATAGCAACAGGAGGCT	
APPV-10F	GTGGCATTCGCATATTTACT	820
APPV-10R	GGCCCCCTTGCTTCATCTAG	

Table S2. Sequence identities between the APPV China/HLJ491/2017 strain in this study and representative APPV strains in GenBank (nt/aa%).

Strain	Genotype	ORF	N^{pro}	C	E^{rns}	E1	E2	P7	NS2	NS3	NS4A	NS4B	NS5A	NS5B
ISDVDL2014016573	1	83.3/92.4	81.1/83.9	83.5/89.2	82.9/93.3	85.3/94.0	86.2/93.4	77.7/92.2	82.5/92.7	85.8/97.7	85.1/94.0	83.5/96.5	80.1/85.6	82.8/91.2
APPV_GER_01	1	83.4/92.2	79.6/82.2	83.5/88.3	83.0/91.9	84.6/93.0	85.5/92.9	79.8/93.8	83.1/93.0	85.6/97.5	82.1/92.5	83.7/96.2	80.3/85.6	83.8/91.9
Ger-NRW_L277	1	83.4/92.3	80.0/82.8	82.3/88.3	83.5/92.4	85.4/94.0	85.5/92.5	80.7/92.2	82.8/93.0	85.8/97.5	82.1/92.5	84.1/96.2	80.0/85.4	83.4/92.0
NL1_Farm1	1	83.3/92.0	81.5/81.1	82.6/85.6	82.2/93.8	86.1/94.5	85.5/92.1	82.4/93.8	82.3/93.3	85.8/96.9	81.6/97.0	83.3/95.9	79.7/85.2	83.0/91.2
AUT-2016_C	1	83.3/91.9	79.6/82.8	83.5/88.3	82.7/91.0	85.6/95.0	85.5/93.8	79.8/90.6	82.5/91.7	84.8/96.9	84.1/92.5	85.3/96.2	80.0/85.4	83.2/91.3
SK68-11	1	83.2/92.3	79.4/82.2	84.4/88.3	81.7/91.9	84.1/94.5	85.6/94.2	80.3/90.6	82.0/92.7	86.7/97.8	81.6/94.0	84.8/96.2	78.7/85.6	83.2/91.5
KU16-6	1	83.0/91.7	80.2/83.3	83.5/89.2	82.1/92.9	84.1/93.5	84.1/92.5	80.8/95.3	83.1/92.0	85.5/96.4	81.1/91.0	84.0/94.1	79.5/86.0	83.1/90.8
China/SWU-KZ/2018	1	83.1/92.1	79.6/81.1	82.6/89.2	81.9/90.5	83.9/94.5	84.8/92.1	81.3/89.1	82.9/91.7	85.6/97.2	84.6/97.0	83.4/96.2	80.8/87.3	83.4/91.2
APPV_GD	1	83.2/91.6	80.9/83.9	82.9/86.5	82.2/91.9	84.4/93.0	85.2/95.4	80.8/90.6	82.5/92.7	84.5/95.8	84.6/91.0	84.0/95.6	80.1/85.0	83.9/90.8
GX01-2018	1	83.5/92.4	78.7/81.7	84.4/91.0	83.0/91.0	84.6/96.0	84.5/93.8	80.3/92.2	84.6/94.3	85.7/97.1	83.6/95.5	84.0/96.5	79.7/85.8	83.8/91.1
GX02-2018	1	83.5/92.4	81.3/82.2	78.4/86.5	81.6/94.8	86.1/94.5	86.4/94.2	83.4/93.8	82.4/93.3	86.2/97.1	83.6/95.5	84.7/96.8	79.7/84.5	83.4/92.0
China/HeNLY/2017	1	82.9/92.2	79.8/82.8	83.5/89.2	82.1/93.3	83.9/94.0	83.8/92.5	82.4/95.3	83.3/92.4	85.3/96.9	80.1/94.0	84.4/95.3	79.2/86.2	83.2/91.3
VIRES_NM01_C1	2	97.6/98.9	95.6/97.2	98.8/100	98.7/99.0	97.2/99.0	97.1/96.7	95.8/100	97.8/99.0	97.5/99.7	96.0/98.5	98.0/99.4	97.8/98.9	97.9/98.9
CH-GD2017	2	93.5/97.3	92.6/93.3	94.6/98.2	92.9/96.2	93.0/95.5	93.1/95.0	91.7/95.3	93.7/98.1	94.1/99.0	94.5/100	93.9/99.1	92.7/96.8	93.5/96.9
GD-YJHSEY3N	2	93.7/97.9	93.1/95.0	94.9/98.2	93.5/98.6	93.5/96.5	93.4/95.4	92.2/96.9	94.2/98.4	94.4/99.7	94.0/100	93.6/99.1	92.8/97.0	93.7/97.7
GD1	2	93.5/97.5	92.8/94.4	94.9/98.2	93.7/98.6	93.1/95.5	93.1/95.0	91.2/93.8	93.7/97.5	94.2/99.4	94.0/100	93.6/98.8	92.6/96.6	93.7/97.3
GD-BH02-2018	2	93.6/97.6	93.0/94.4	94.9/99.1	93.5/98.1	93.5/96.5	93.4/95.0	91.7/95.3	94.1/98.1	94.1/99.4	94.5/100	93.6/98.8	92.5/96.6	93.8/97.6
China/SWU-MY/2018	2	93.5/97.4	92.2/92.8	94.9/97.3	94.1/98.6	92.5/96.5	93.6/96.3	91.7/93.8	93.0/97.5	94.2/99.1	95.0/100	93.7/98.2	92.1/96.4	94.1/97.6
China/GD-SD/2016	2	93.3/97.1	92.6/93.9	94.6/96.4	92.9/96.2	91.5/95.5	92.0/94.2	92.2/100	93.6/97.5	93.9/99.1	96.0/98.5	93.2/98.2	92.6/96.8	94.0/97.1
GX02-2019	2	93.5/97.4	92.6/93.3	94.6/98.2	93.5/97.1	92.6/96.5	93.4/94.6	89.6/98.4	94.7/97.1	94.1/99.3	91.0/97.0	93.8/98.8	92.3/96.6	94.1/97.5
GD-ZW-2017.10	3	81.0/91.1	78.0/81.7	81.7/91.9	80.6/93.3	80.4/93.0	80.6/91.3	78.8/89.1	81.0/91.7	84.7/96.7	77.6/91.0	80.9/95.6	77.6/83.3	81.2/89.9
AH-GL-2017.04	3	80.9/91.1	77.6/81.7	81.4/91.9	79.8/93.3	80.2/93.5	80.6/90.0	78.8/90.6	80.7/91.1	84.8/96.8	77.6/91.0	80.9/95.6	77.9/83.7	81.4/89.6
GD-MH01-2018	3	80.8/91.1	77.8/82.2	81.7/91.9	80.0/92.9	80.6/93.5	80.8/92.5	78.2/90.6	80.4/91.1	84.3/96.4	77.6/91.0	80.5/95.0	77.5/83.7	81.3/89.6
Annaka/2020	3	80.9/90.9	76.7/82.8	82.3/91.9	80.2/91.0	81.2/92.0	80.5/90.5	78.2/92.2	81.3/89.5	83.0/96.9	81.1/91.0	81.8/95.6	77.5/83.3	82.0/90.1
